# Prognostic value of lymphocyte subsets in diffuse large B cell lymphoma

**DOI:** 10.3389/fonc.2025.1671062

**Published:** 2025-10-31

**Authors:** Lihua Qiu, Lijing Xing, Meng Zhao, Biwen Sun, Xinpei Zhou, Min Li, Guoguang Ying, Huilai Zhang, Chen Tian

**Affiliations:** ^1^ Tianjin Medical University Cancer Institute and Hospital, National Clinical Research Center for Cancer, Key Laboratory of Cancer Prevention and Therapy, Tianjin’s Clinical Research Center for Cancer, Tianjin, China; ^2^ School of Medical Technology, Tianjin Medical University, Tianjin, China; ^3^ Laboratory of Cancer Cell Biology, Tianjin Medical University Cancer Institute and Hospital, National Clinical Research Center for Cancer, Tianjin, China

**Keywords:** diffuse large B-cell lymphoma, lymphocyte subsets, CD4+ T cells, CD8+ T cells, CD4+/CD8+ ratio

## Abstract

**Introduction:**

Although some clinical prognostic parameters and gene expression features have been identified to be associated with the prognosis of diffuse large B cell lymphoma (DLBCL), clinical outcomes of DLBCL remains unpredictable. Lymphocyte subpopulations are considered to be prognostic factors for DLBCL, however due to the small sample size, conflicting views exist.

**Methods:**

301 newly diagnosed DLBCL patients treated at our center from January 2015 to December 2019 were retrospectively analyzed to explore the relationship of lymphocyte subsets and prognosis prediction.

**Results:**

In this retrospective single-center study, patients with more severe disease, defined by either advanced Ann Arbor stage or a high-risk IPI score, consistently exhibited significantly lower percentages of CD19+ B cells, CD4+ T cells and CD4+/CD8+ ratio, while percentage of CD8+ T cells were significantly higher. There were no significant differences in percentages of CD3+ T cells and CD16+CD56+ NK cells. Correlation analysis revealed significant associations between lymphocyte subsets and clinical features such as Ann Arbor stage, IPI score, lactate dehydrogenase (LDH), beta-2 microglobulin (b2-MG), Ki-67, age, and neutrophils. Higher percentages of CD19+ B cells, CD4+ T cells, and CD16+CD56+ NK cells, a higher CD4+/CD8+ ratio, and a lower percentage of CD8+ T cells at diagnosis predicted good response after R-CHOP treatment. Multivariate analysis indicated that a low CD4+/CD8+ ratio was independently associated with worse PFS (p=0.049) and showed a trend toward worse OS (p=0.086). Patients were dichotomized into high and low groups using the median value of CD4+/CD8+ ratio, Kaplan-Meier analysis showed that patients with CD4+/CD8+ ratio ≥1.19 had significantly longer PFS and OS compared to those with CD4+/CD8+ ratio <1.19.

**Discussion:**

Lymphocyte subsets, especially CD4+/CD8+ ratio, could be recommended as a potential prognostic indicator for DLBCL.

## Introduction

Diffuse large B-cell lymphoma (DLBCL) is the most common type of adult non-Hodgkin lymphoma (NHL), accounting for 30% to 40% of all NHL cases, which represents a high heterogeneity in histological, immunohistochemical, and molecular characteristics (1–3). Prognosis assessment of DLBCL largely relies on traditional clinical-pathological scoring systems, such as the International Prognostic Indexs (IPI) (4), which indices mainly reflect tumor burden and basic clinical conditions but fail to fully elucidate the role of the immune system in tumor progression. Therefore, exploring novel biomarkers and immune indicators is of significant clinical importance.

The host immune system plays a crucial role in anticancer defense, and its dysfunction is closely associated with tumor development and progression (5). The success of cancer immunotherapy suggests that analyzing the host’s systemic immune response is essential for assessing the prognosis of DLBCL (6). Previous studies have demonstrated that immune deficiency is closely linked to the pathogenesis and prognosis of lymphoma. For example, in mouse models, the critical role of percentage of CD8+ T cells in preventing lymphoma has been established (7). Patients with a higher percentage of peripheral blood lymphocytes tend to have a better prognosis compared to those with lower percentages of peripheral blood lymphocytes (8). The percentage of CD4+ T cells are generally considered beneficial, and a reduction in the percentage of CD4+ T cells is closely associated with poor treatment response and prognosis in DLBCL patients (9). In contrast, an increase in the percentage of CD8+ T cells is associated with poor prognosis in DLBCL patients (10). Natural Killer (NK) cells, as an essential component of the innate immune system, play a key role in immune surveillance (11, 12). Changes in the CD4+/CD8+ ratio are significant in immune surveillance across various cancers, yet their specific role in DLBCL has not been fully explored (13). Lymphocyte subpopulations are considered to be prognostic factors for DLBCL; however, due to the relatively small sample sizes in previous studies (e.g., Berthelot et al. (14) with n=53; Xu et al. (15) with n=55), conflicting views exist. This study aims to investigate the relationship between lymphocyte subsets and prognosis in DLBCL patients, assess the potential of the CD4+/CD8+ ratio as an immunological prognostic marker, and further reveal the connection between the immune system and treatment outcomes. The goal is to provide a theoretical foundation for the development of innovative therapeutic strategies to improve the clinical prognosis of DLBCL patients.

## Materials and methods

### Patients

**Figure 1:**
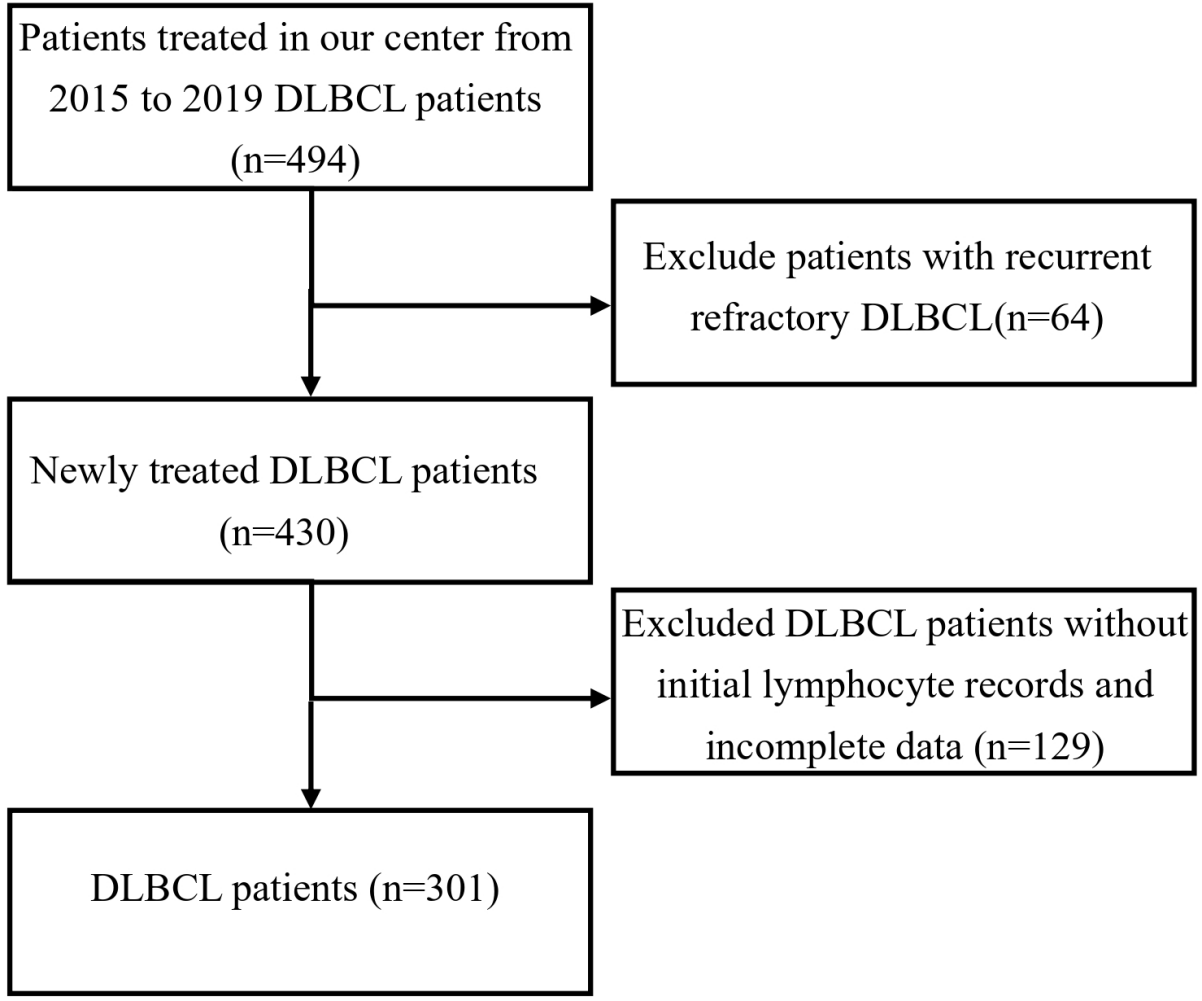
Patient enrollment screening flow chart.

This study is a retrospective analysis that included 301 patients diagnosed with DLBCL between January 2015 and December 2019 at our institution (**Figure 1**). Inclusion criteria: patients with *de novo*, newly diagnosed diffuse large B-cell lymphoma (DLBCL); patients with a history of prior lymphoma or those with histologically transformed disease were excluded. Exclusion criteria: patients with known human immunodeficiency virus (HIV) infection or other severe immunodeficiency states were excluded. Among these, 143 were female and 158 were male. According to the Ann Arbor staging system, 136 patients were in stages I/II, and 165 patients were in stages III/IV. The baseline characteristics of the patients are summarized in **Table 1**. A total of 301 patients were treated with the R-CHOP regimen. Among them, 258 patients had complete follow-up data available for subsequent analysis. Furthermore, 213 patients had at least four cycles of continuous lymphocyte subset testing data. Baseline peripheral blood samples (T0, at diagnosis) were collected for lymphocyte subset analysis, and subsequent tests were performed after two (T2) and four (T4) cycles of treatment. This study was approved by the Research Ethics Committee of Tianjin Medical University Cancer Institute and Hospital with informed consent waiver granted. The study adhered to the principles of the Declaration of Helsinki as set forth by the World Medical Association.

**Table 1:** Characteristics of DLBCL patients at diagnosis.

Characteristics	Values
Number of patients	301
Age	
< 60	140(46.5%)
≥ 60	161(53.5%)
Sex	
male	158(52.5%)
female	143(47.5%)
NEUT(*10^9/L)	3.8(2.7,5.3)
GLO(g/L)	30.8(28.1,34.1)
β2-MG(U/L)	2.2(1.7,3.0)
LDH(U/L)	219.0(178.0,331.5)
KI-67	
< 80	128(42.5%)
≥ 80	174(57.8%)
Ann Arbor stage	
I-II	136(45.2%)
III-IV	165(64.8%)
IPI score	
0-2	203(67.4%)
3-5	98(32.6%)

Data are presented as M(IQR) or numbers (%).

### Detection of lymphocyte subsets by flow cytometry

The percentages of T-cell subsets (including CD3+ T cells, CD4+ T cells, CD8+ T cells), CD19+ B cells, and CD16+CD56+ NK cells in peripheral blood were measured by DxFLEX Flow Cytometer (DxFLEX) using the corresponding antibodies (All purchased from Beckman Coulter, Inc.). The procedure was as follows: After overnight fasting, 5 mL of peripheral blood was collected from patients in the morning using Ethylenediaminetetraacetic Acid (EDTA) anticoagulation tubes, ensuring that the samples were free of hemolysis and clotting. The samples were labeled, and T, NK, and B cell subsets were marked. Antibody panels were selected based on experimental requirements. Standard amounts of antibody reagents (10 μL) were added to the bottom of the tube, followed by the addition of 100 μL of mixed anticoagulated whole blood, taking care not to smear the walls of the tube to avoid false readings. The mixture was gently vortexed and incubated at room temperature (20-25 °C) in the dark for 15–20 minutes. Then, 0.5 mL of Lysing Solution (1x) was added to lyse the red blood cells, followed by gentle vortexing and incubation for 10 minutes until the red blood cells were fully lysed. Next, 0.5 mL of Phosphate-Buffered Saline (PBS) was added, and the solution was vortexed before being incubated at room temperature for 5 minutes. The sample was centrifuged at 1000 rpm for 3 minutes. This step was repeated once more, followed by adding 0.5 mL PBS, vortexing, and preparing the sample for flow cytometry analysis.

### Statistical analyses

Descriptive statistics were expressed as frequency (percentage) or median (range). Progression-free survival (PFS) was defined as the time from the DLBCL diagnosis to the documented disease progression or death, with follow-up data censored at the last disease assessment. Overall survival (OS) was defined as the time from DLBCL diagnosis to death from any cause, with follow-up data censored at the last follow-up visit. The DLBCL diagnosis date was considered the date of histological confirmation.

Progression-free survival (PFS) and overall survival (OS) were estimated using the Kaplan-Meier method and compared with the Log-rank test.

The choice of statistical test was determined by the distribution characteristics of the data. Continuous variables that were normally distributed were analyzed using one-way analysis of variance (ANOVA) for group comparisons. For continuous variables that deviated from a normal distribution, non-parametric tests were employed: the Mann-Whitney U test for two-group comparisons and Spearman’s correlation analysis to explore the relationships between lymphocyte subsets and clinical characteristics. Cox proportional hazards models were employed for both univariate and multivariate analyses, to construct the multivariate model, variables with p< 0.05 in the univariate analysis or those deemed clinically relevant were included in the multivariate model. We further assessed multicollinearity among these variables before finalizing the model. Statistical significance for identifying independent prognostic factors in the multivariate analysis was set at p < 0.05. Cox proportional hazards regression was used to identify independent prognostic factors, and the proportional hazards assumption was verified for each variable using Schoenfeld residuals. Given the exploratory nature of the correlation and subgroup analyses, p-values are reported without adjustment for multiple comparisons. The findings should therefore be interpreted as generating hypotheses for future validation.

For the analysis of dynamic changes in lymphocyte subsets, which included a subset of 213 patients with serial measurements, missing data at one or more time points were handled by listwise deletion. Only patients with complete data across all analyzed time points were included in the longitudinal analysis.

*Post Hoc* Power Analyses: given the retrospective nature of this study, a prior sample size calculation was not performed. However, *post hoc* power analyses were conducted to quantify the reliability of our key findings. For survival outcomes: The analysis for the primary multivariable association (a high CD4+/CD8+ ratio with better PFS, HR = 0.204) achieved a power of >99.9%. A sensitivity analysis for a smaller effect (HR = 0.5) still yielded a power of >95%. For treatment response: The analysis for the association between a high baseline CD4+/CD8+ ratio and achieving Complete Remission (CR) in the evaluable subgroup (n=213) also demonstrated high robustness, achieving a power of 99.6%. A sensitivity analysis assuming a more conservative effect (OR = 3.0) confirmed a power of 95%.

Flow cytometry data were analyzed using FlowJo software (version 10.8.1, Tree Star). All statistical analyses were conducted using IBM SPSS Statistics (version 25) and GraphPad Prism (version 10.0.2), with *p* < 0.05 considered statistically significant.

## Results

### The relationship between lymphocyte subsets and clinical staging in DLBCL patients

**Figure 2:**
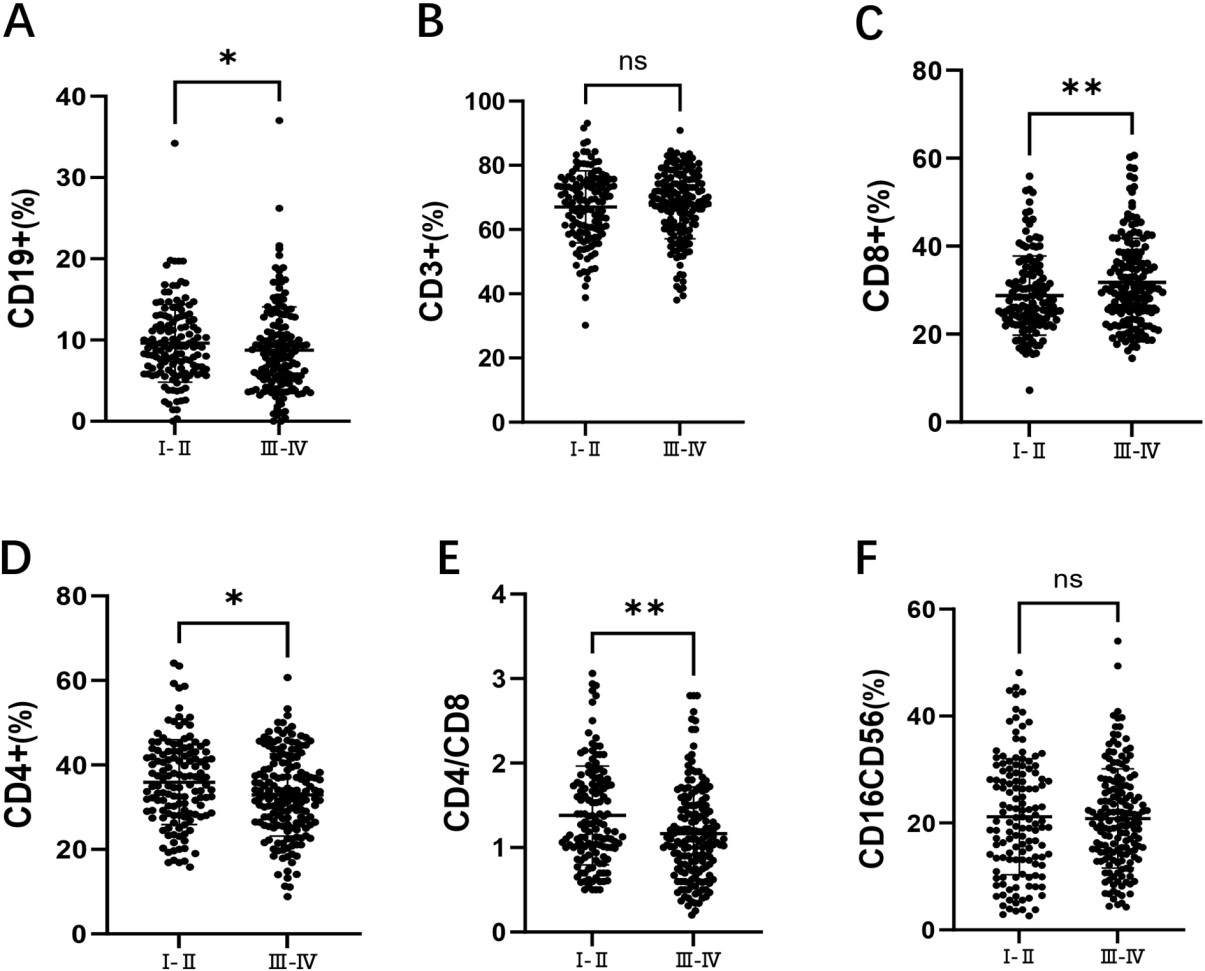
Comparison of lymphocyte subsets in DLBCL patients at stages I/II and III/IV. **(A)** the percentage of CD19+ B cells in DLBCL patients at stages I/II and III/IV. **(B)** the percentage of CD3+ T cells in DLBCL patients at stages I/II and III/IV. **(C)** the percentage of CD8+ T cells in DLBCL patients at stages I/II and III/IV. **(D)** the percentage of CD4+ T cells in DLBCL patients at stages I/II and III/IV. **(E)** the CD4+/CD8+ ratio in DLBCL patients at stages I/II and III/IV. **(F)** the percentage of CD16+CD56+ NK cells in DLBCL patients at stages I/II and III/IV. Error bars represent the mean ± standard deviation (SD). * *p* < 0.05;** *p* < 0.01; *** *p <* 0.001; **** *p <* 0.001.

Among the 301 patients, 136 were in stages I/II, and 165 were in stages III/IV. As shown in **Figure 2A**, compared to early-stage patients, those in the advanced stages had a significantly lower of CD19+ B cell percentages(9.20% vs 8.20%, *p* = 0.046). As shown in **Figure 2B**, there was no significant difference in the percentage of CD3+ T cells between the two groups. In **Figure 2C**, the percentage of CD8+ T cells was significantly higher in advanced-stage patients (30.00% vs 26.40%, *p* = 0.009). As shown in **Figure 2D**, the percentage of CD4+ T cells was significantly lower in advanced-stage patients compared to early-stage patients (32.60% vs 35.24%, *p* = 0.019). **Figure 2E** shows that the CD4+/CD8+ T cell ratio was significantly reduced in advanced-stage DLBCL patients (1.10 vs 1.28, *p* = 0.001). Finally, as shown in **Figure 2F**, there was no significant difference in the percentage of CD16+CD56+ NK cells between the two groups.

### The relationship between lymphocyte subsets and risk stratification in DLBCL patients

**Figure 3:**
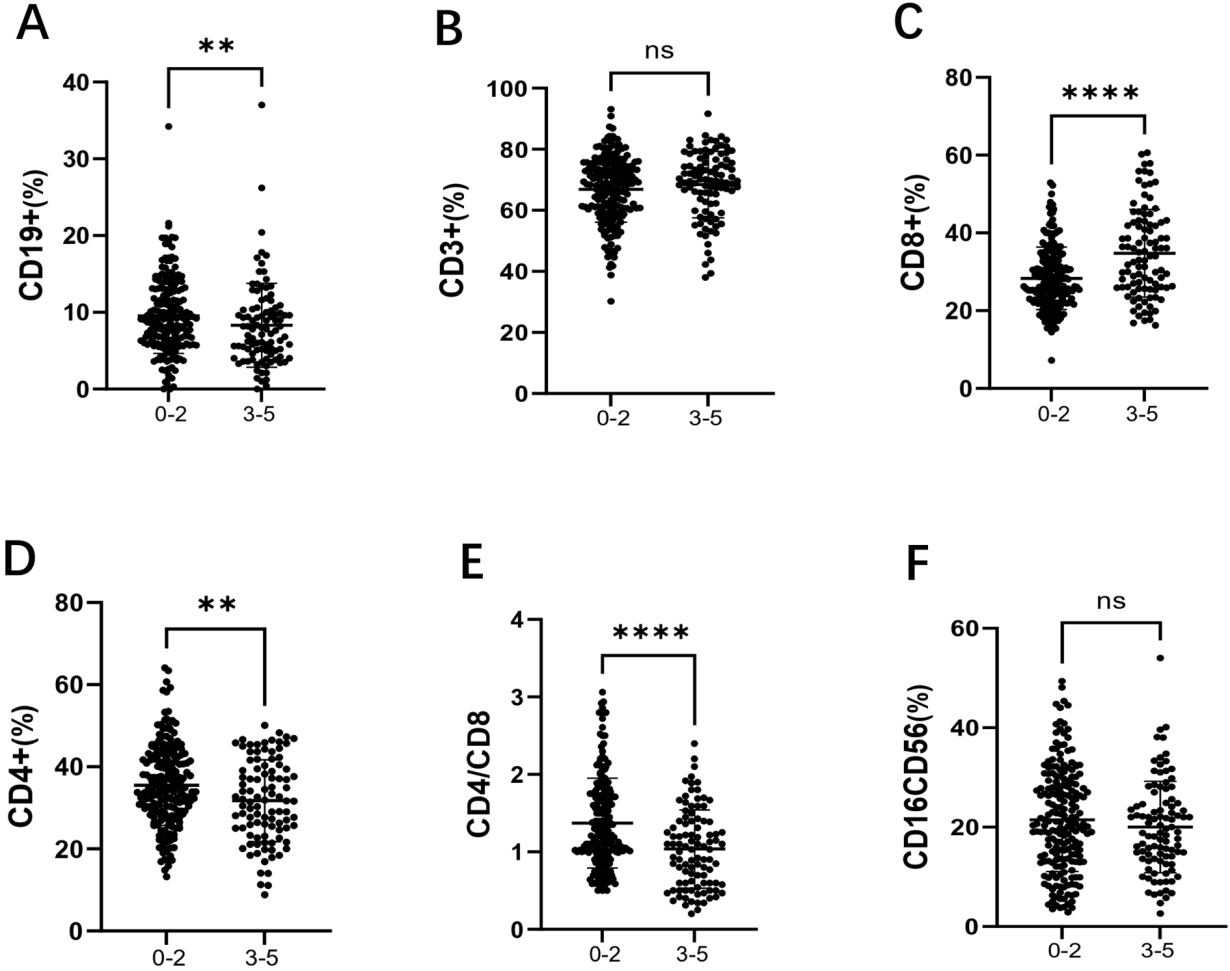
Comparison of lymphocyte subsets in the low-risk group (IPI 0-2) and high-risk group (IPI 3-5) in DLBCL patients. **(A)** the percentage of CD19+B cells in DLBCL low-risk and high-risk groups; **(B)** the percentage of CD3+ T cells in DLBCL low-risk and high-risk groups; **(C)** the percentage of CD8+ T cells in DLBCL low-risk and high-risk groups; **(D)** the percentage of CD4+ T cells in DLBCL low-risk and high-risk groups; **(E)** the CD4+/CD8+ ratio in DLBCL low-risk and high-risk groups; **(F)** the percentage of CD16+CD56+ NK cells in DLBCL low-risk and high-risk groups. Error bars represent the mean ± SD. * *p* < 0.05;** *p* < 0.01; *** *p* < 0.001; **** *p* < 0.001.

Among the 301 patients, 203 were in the low-risk group (IPI 0-2) and 98 were in the high-risk group (IPI 3-5). As shown in **Figure 3A**, the percentage of CD19+ B cells was significantly lower in the high-risk group compared to the low-risk group (7.70% vs 9.10%, *p* = 0.009). As shown in **Figure 3B**, there was no significant difference in the percentage of CD3+ T cells between the two groups. As shown in **Figure 3C**, the percentage of CD8+ T cells was significantly higher in the high-risk group (33.50% vs 27.10%, *p* < 0.001). As shown in **Figure 3D**, the percentage of CD4+ T cells was significantly lower in the high-risk group compared to the low-risk group (31.65% vs 34.60%, *p* = 0.007). As shown in **Figure 3E**, the CD4+/CD8+ T cell ratio was significantly reduced in the high-risk group (1.03 vs 1.22, *p* < 0.001). Finally, as shown in **Figure 3F**, there was no significant difference in the percentage of CD16+CD56+ NK cells between the two groups.

### Lymphocyte subsets are correlated with clinical features such as Ann Arbor stage, IPI, LDH, β2-microglobulin, KI-67, age, and NEUT

Correlation analysis (**Table 2**) shows the associations between baseline levels of various lymphocyte markers (such as CD19+B cells, CD3+ T cells, CD8+ T cells, etc.) and multiple clinical indicators. The table lists the correlation coefficients (r values) between each lymphocyte subset and different clinical features, with statistical significance indicated (* *p* < 0.05, ** *p* < 0.01).

**Table 2:** Correlation analysis between baseline (T0) lymphocyte subsets and clinical characteristics.

Subsets	Ann Arbor stage	IPI	LDH	β	KI-67	Age	NEUT
CD19+	-0.078	-0.135**	-0.087*	-0.140**	0.009	-0.120**	-0.119**
CD3+	0.010	0.028	0.005	0.011	0.022	0.039	-0.047
CD8+	0.099*	0.150**	0.096*	0.120**	0.059	0.083*	0.013
CD4+	-0.106*	-0.111**	-0.101**	-0.063	-0.113**	0.021	-0.090*
CD4+/CD8+	-0.128**	-0.158**	-0.129**	-0.104**	-0.103**	-0.023	-0.064
CD16+CD56+	0.003	-0.031	-0.015	0.007	-0.056	-0.004	0.051

* *p* < 0.05,** *p* < 0.01

Regarding the correlation with the percentage of CD19+B cells, significant negative correlations were observed between the percentage of CD19+B cells and IPI score, LDH, β2-microglobulin, age, and NEUT (mostly p < 0.01). This suggests that, in certain cases, the percentage of CD19+B cell levels may be associated with tumor progression in DLBCL, particularly in cases of immune dysfunction or advanced disease. As for the percentage of CD3+ T cells, while correlations with clinical features were generally weak, this indicates that the percentage of CD3+ T cells may not play a significant role in these specific clinical markers. The percentage of CD8+ T cells, on the other hand, showed positive correlations with Ann Arbor stage, IPI score, LDH, β2-microglobulin, and age, suggesting that the percentage of CD8+ T cell counts may play an important role in the progression or treatment response of DLBCL. The percentage of CD4+ T cells were negatively correlated with Ann Arbor stage, IPI score, LDH, KI-67, and NEUT, implying that CD4+ T cell levels may play a crucial role in DLBCL progression, related infections, or treatment responses. The CD4+/CD8+ ratio was significantly negatively correlated with multiple clinical indicators, including Ann Arbor stage, IPI score, LDH, β2-microglobulin, and KI-67, suggesting that changes in this ratio could serve as an important immune monitoring marker reflecting disease progression and prognosis. Finally, The percentage of CD16+CD56+ NK cells showed little correlation with clinical indicators, indicating that this cell population may not serve as a significant immune monitoring marker in this particular disease model.

In summary, an increase in the percentage of CD8+ T cells is associated with poor prognosis (such as high IPI scores and elevated LDH levels), while higher levels of the percentages of CD4+/CD8+ ratio, CD4+ T cells, and CD19+B cells are linked to better prognosis. These findings suggest that lymphocyte subsets may play an important role in immune surveillance and clinical prognosis in DLBCL.

### The prognostic value of lymphocyte subsets in DLBCL patients

A total of 213 patients received at least four cycles of R-CHOP treatment and had corresponding lymphocyte subset data available. Among them, 70 patients (32.9%) achieved Complete Remission (CR). The patients were divided into two groups: the CR group and the non-CR group.

First, we investigated the predictive value of baseline (T0) immune profiles. At diagnosis, the CR group had a slightly higher percentage of CD19+ B cells than the non-CR group (p = 0.044). Notably, the percentage of CD8+ T cells was significantly lower in the CR group (p = 0.002), whereas the percentages of CD4+ T cells (p < 0.001) and CD16+CD56+ NK cells (p < 0.001) were significantly higher. Consequently, the baseline CD4+/CD8+ ratio was significantly elevated in the CR group (p < 0.001).

Furthermore, we assessed whether this predictive effect was consistent across IPI risk groups. We tested for an interaction between the baseline CD4+/CD8+ ratio and IPI risk group to determine if the effect of the ratio on treatment response differed by risk category. The interaction term was not statistically significant (*p* = 0.998), indicating that the positive association between a higher CD4+/CD8+ ratio and an increased likelihood of achieving Complete Remission was consistent and did not differ between patients with low-risk and high-risk IPI scores.

**Figure 4:**
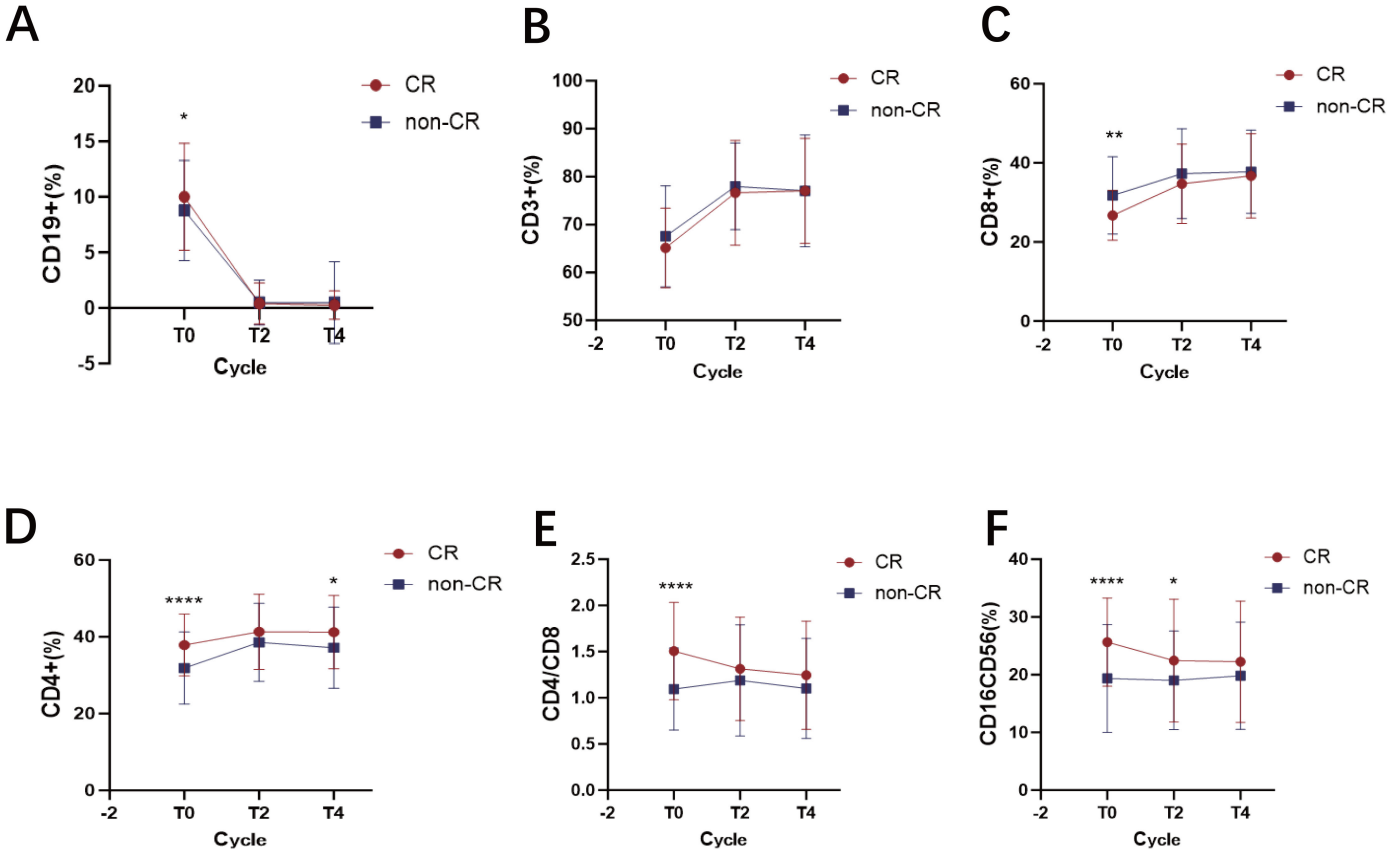
Dynamic changes in lymphocyte subsets in DLBCL patients achieving CR versus non-CR after R-CHOP therapy. **(A)** the percentage of CD19+ B cells at diagnosis and dynamic changes after treatment in the CR and non-CR groups. **(B)** the percentage of CD3+ T cells at diagnosis and dynamic changes after treatment in the CR and non-CR groups. **(C)** the percentage of CD8+ T cells at diagnosis and dynamic changes after treatment in the CR and non-CR groups. **(D)** the percentage of CD4+ T cells at diagnosis and dynamic changes after treatment in the CR and non-CR groups. **(E)** the CD4+/CD8+ ratio at diagnosis and dynamic changes after treatment in the CR and non-CR groups. **(F)** the percentage of CD16+CD56+ NK cells at diagnosis and dynamic changes after treatment in the CR and non-CR groups. Data points represent the group mean at each time point for CR (n=70) and non-CR (n=143) patients, respectively. Lines connect the mean values at consecutive time points to illustrate the overall trend for each group. Error bars represent the mean ± SD. * *p* < 0.05; ** *p* < 0.01; **** *p <*0.0001.

We next monitored the dynamic changes of these subsets during treatment. As expected, by T2, the percentage of CD19+ B cells in the CR group decreased by 95.13%, while in the non-CR group, it decreased by 94.46%. This change is associated with the targeted action of rituximab (R). Subsequently, the percentages remained stable in both groups (**Figure 4A**). From T0 to T4, the percentages of CD3+ T cells (**Figure 4B**) and CD8+ T cells (**Figure 4C**) gradually increased with immunochemotherapy, reaching a plateau at T4. For CD4+ T cells (**Figure 4D**), a similar increasing trend was observed; importantly, at all time points, the percentage in the CR group was consistently higher than that in the non-CR group, and this difference remained statistically significant at T4 (p = 0.014). Consequently, the CD4+/CD8+ ratio was consistently higher in the CR group than in the non-CR group at all time points, although the ratio gradually decreased in both groups with treatment progression (**Figure 4E**). Finally, the percentage of CD16+CD56+ NK cells gradually decreased during the treatment process in both groups (**Figure 4F**), which may be related to Antibody-Dependent Cell-Mediated Cytotoxicity (ADCC), leading to NK cell depletion.

### Patient survival

**Figure 5:**
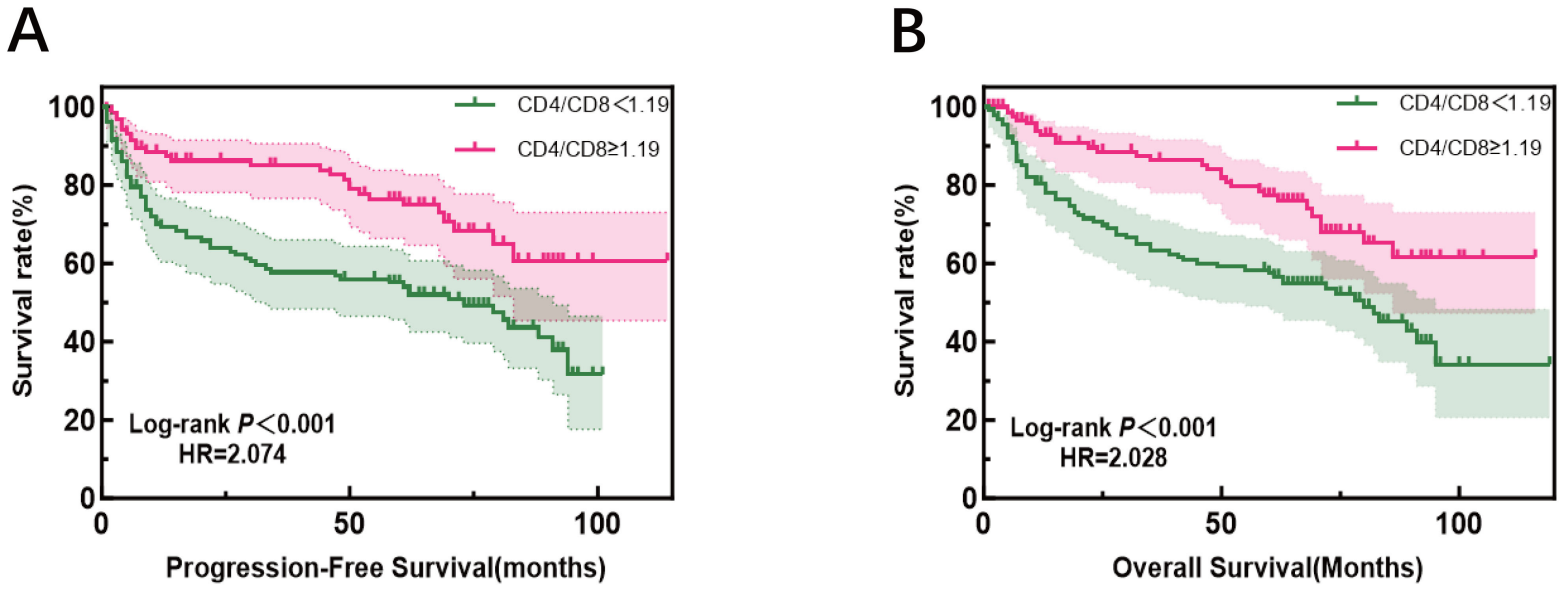
Progression-free survival (left) and overall survival (right) according to CD4+/CD8+ratio, patients were divided into high- and low-ratio groups based on the median value (1.19) of the entire cohort.

It is important to clarify the distinct patient cohorts used for different analyses to avoid potential confusion. The evaluation of treatment response (Complete Remission, CR) was performed in a subset of 213 patients who received at least four cycles of R-CHOP and had serial biomarker data. In contrast, the survival analysis (PFS and OS), including the 10-year overall survival rate, was based on a larger cohort of 258 patients with complete follow-up data, which encompasses a broader range of clinical outcomes beyond initial treatment response. The median follow-up for the cohort was 96.0 months (range, 60.0-120.0 months). At the data cutoff (December 2024), the median PFS was 88.0 months (95% CI, 78.0–98.0), while the median OS was not reached (NR). The observed OS rate at the 10-year time point was 63.6%. In univariate analysis, age ≥60 years, high Ann Arbor stage, elevated LDH levels, high IPI score, and a high percentage of CD8+ T cells were all associated with worse PFS. Conversely, a low percentage of CD4+ T cells and low CD4+/CD8+ ratio were also typically associated with poorer PFS. Similarly, age ≥60 years, high Ann Arbor stage, elevated LDH levels, high IPI score, and a high percentage of CD8+ T cells were associated with poorer OS, while low CD4+ T cells percentage and low CD4+/CD8+ ratio were also associated with worse OS (**Figure 5**, **Table 3**).

**Table 3:** PFS and OS prognostic factors.

Variable	PFS			OS		
	Univariate	Multivariate	HR(95%CI)	Univariate	Multivariate	HR(95%CI)
Age (< 60/≥ 60)	0.001	0.285	1.334(0.786,2.263)	0.002	0.221	1.393(0.819,2.367)
Sex (male/female)	0.675			0.754		
Ann Arbor stage (I-II/III-IV)	<0.001	0.626	0.856(0.460,1.596)	0.001	0.504	0.806(0.428,1.518)
NEUT	0.280			0.439		
GLO	0.373			0.432		
β2-MG	0.698			0.710		
KI-67	0.211			0.104		
LDH	<0.001	0.064	1.001(1.000,1.001)	<0.001	0.020	1.001(1.000,1.001)
IPI	<0.001	0.011	1.382(1.078,1.770)	<0.001	0.028	1.324(1.031,1.699)
CD19+	0.054	0.582	0.987(0.941,1.034)	0.041	0.413	0.981(0.937,1.027)
CD3+	0.900			0.875		
CD8+	<0.001	0.257	0.973(0.928,1.020)	<0.001	0.349	0.977(0.932,1.025)
CD4+	0.001	0.303	1.030(0.974,1.088)	0.001	0.494	1.019(0.966,1.077)
CD4+/CD8+	<0.001	0.049	0.204(0.042,0.994)	<0.001	0.086	0.253(0.053,1.216)
CD16+CD56+	0.293			0.267		

In multivariate Cox regression analysis, elevated LDH showed a trend toward significance with worse PFS [*p* = 0.064, HR = 1.001 (1.000, 1.001)] and with worse OS [*p* = 0.020]. Similarly, a high IPI score was associated with worse PFS [*p* = 0.011, HR = 1.382 (1.078, 1.770)] and worse OS [*p* = 0.028].

More interestingly, compared to patients with a low ratio, those with a high CD4+/CD8+ ratio had a significantly reduced risk of progression or death [*p* = 0.049, HR = 0.204 (0.042-0.994), equivalent to an 80% risk reduction. A similar protective trend was observed for OS [p=0.086]. Assessment of the Schoenfeld residuals confirmed that the proportional hazards assumption was not violated.

These results suggest that age, Ann Arbor stage, LDH levels, IPI score, the percentages of CD8+ T cells, CD4+ T cells, and the CD4+/CD8+ ratio significantly impact PFS and OS. In multivariate analysis, LDH and IPI scores remained strong prognostic factors, while the CD4+/CD8+ ratio as a prognostic factor is particularly noteworthy. Other factors, such as gender, NEUT, GLO, β2-MG, KI-67, the percentages of CD3+ T cells, and CD16+CD56+ NK cells, did not show a significant impact on PFS or OS. Further Kaplan-Meier analysis demonstrated that patients with a CD4+/CD8+ ratio ≥1.19 had significantly better PFS and OS compared to those with a ratio <1.19. This result strengthens the view of the CD4+/CD8+ ratio as a potential biomarker for immune status evaluation and predicting treatment outcomes.

## Discussion

The immune system plays a crucial role in cancer prevention and control, especially in their immune surveillance and cytotoxic functions (16, 17). This study establishes that changes in lymphocyte subsets, especially the CD4+/CD8+ ratio, are significantly associated with clinical features, treatment response, and survival outcomes, highlighting its prognostic value in DLBCL.

The observed decline in CD19+ B cells, CD4+ T cells (18), and the CD4+/CD8+ ratio, coupled with an increase in CD8+ T cells in high-risk (IPI 3-5) and advanced-stage (III/IV) patients, points to a state of progressive immune suppression within the DLBCL tumor microenvironment. The association between a favorable immune constitution—characterized by higher CD19+ B cells, CD4+ T cells (9), NK cells, and a higher CD4+/CD8+ ratio (10)—and an increased likelihood of achieving CR underscores the critical link between baseline immune status and treatment efficacy. Elevations in CD19+ B and CD4+ T cells (18) may facilitate more effective anti-tumor immunity, whereas an increased CD8+ T cell percentage could reflect the activation of immune evasion pathways.

Survival analysis confirmed the prognostic power of these immune parameters. The favorable impact of high CD19+ B cells, CD4+ T cells (8, 9, 19), and the CD4+/CD8+ ratio, along with the adverse association of elevated CD8+ T cells (10) with outcome, aligns with findings in other cancers where such an immune profile indicates tumor-induced immunosuppression (20). Notably, the correlation between the CD4+/CD8+ ratio and survival emphasizes its potential as a prognostic biomarker.

NK cells, marked by CD16+CD56+, did not show significant differences between early and late-stage or high-risk and low-risk groups, suggesting that NK cells may play a relatively limited role in the immune landscape of DLBCL in this cohort. The elevated NK cell levels observed in CR patients may be attributed to their role in antibody-dependent cellular cytotoxicity (ADCC), a key mechanism of rituximab (4). The gradual decline in NK cell levels after treatment further supports this hypothesis, indicating that NK cells may be exhausted as they perform their cytotoxic functions.

The CD4+/CD8+ ratio not only reflects the balance of immune responses but may also reveal the extent of immune evasion in the tumor microenvironment. A lower CD4+/CD8+ ratio was significantly associated with worse PFS, and showed a trend toward association with worse OS (p=0.086). This finding is consistent with several studies suggesting that an imbalance in T cell subsets may indicate poor clinical prognosis in hematologic malignancies, particularly lymphoma (21). Previous research has indicated that circulating CD4+ and CD8+cells can serve as predictive or prognostic biomarkers for hematologic diseases. For example, Lu Y et al. defined an immune clinical prognostic index for newly diagnosed follicular lymphoma patients receiving R-CHOP chemotherapy, where blood CD4+ and CD8+ T cell counts were closely associated with patient prognosis (22). Furthermore, studies have found that a lower CD4+/CD8+ ratio at diagnosis is linked to poor prognosis in patients with multiple myeloma and Waldenström’s macroglobulinemia (23, 24). Evidence from other lymphomas supports the prognostic role of T-cell subsets: in mantle cell lymphoma, low CD4+ T cell counts have been linked to poor prognosis (25). Similarly, in the study of mycosis fungoides, Yi An et al. found that the CD4+/CD8+ ratio in peripheral blood was an independent predictor of patients’ response to total skin electron beam therapy, with higher CD4+/CD8+ ratios correlating with poorer responses (26). However, there have been differing views on the role of the CD4+/CD8+ ratio in the prognosis of DLBCL patients, and relevant research remains insufficient (14, 15). Our study strengthens the evidence for the CD4+/CD8+ ratio as a robust prognostic marker in DLBCL and extends its relevance across different clinical stages and risk stratifications.

The observed long-term overall survival rate of 63.6% at 10 years, which appears favorable relative to the CR rate of 32.9%, is clinically plausible and can be explained within the context of frontline R-CHOP therapy. It is critical to distinguish between the depth of initial response and the durability of disease control. Patients who achieved a PR or even those with stable disease (SD) could still derive significant and prolonged clinical benefit from R-CHOP, contributing to long-term survival. Furthermore, a single cycle of chemotherapy can sometimes effectively ‘downstage’ the disease and alleviate tumor-related symptoms for an extended period, even without achieving a formal CR. This phenomenon underscores that long-term survival is not exclusively dependent on achieving CR but also on achieving sustained disease control with the initial therapy.

A key implication of our work is that the CD4+/CD8+ ratio could be translated into a practical tool to refine the IPI, potentially leading to more personalized treatment strategies. The prospective validation of this integrated biomarker is now essential to advance its clinical translation.

Despite these insights, our study has several limitations. We acknowledge that the use of percentages could be influenced by overall lymphopenia, and future studies should explore absolute cell counts. Furthermore, we acknowledge that potential confounders, such as concurrent infections or prior treatments, were not fully adjusted for in our analysis, which may influence the interpretation of the results. Beyond the parameters measured in this study, the systemic immune landscape in DLBCL is complex. The role of other key players, such as Myeloid-Derived Suppressor Cells (MDSCs) and their suppressive effects on T-cell function, or the profile of circulating cytokines, were not investigated. Incorporating these measures in the future would provide a more comprehensive understanding of the immune context and help to better elucidate the biological mechanisms underlying the prognostic power of the CD4+/CD8+ ratio. Additionally, as a single-center retrospective analysis with a relatively small sample size, our findings require confirmation through large-scale, multi-center prospective studies.

Looking forward, several promising research directions emerge from our findings. Most importantly, the findings from this single-center investigation warrant validation in large-scale, multi-center cohorts. First, future studies should investigate the relationship between lymphocyte subsets and DLBCL molecular subtypes (e.g., germinal center B-cell-like [GCB] and activated B-cell-like [ABC]), which could reveal subtype-specific immune profiles and provide deeper insights into the tumor-immune microenvironment. Second, with the growing use of immunotherapies such as immune checkpoint inhibitors and CAR-T, it will be crucial to explore how pre-treatment lymphocyte subsets and their dynamic changes predict response to these novel therapies. This could help identify biomarkers for patient selection and strategies for combining or sequencing immunotherapy with conventional regimens.

In summary, our study demonstrates that lymphocyte subsets, particularly the CD4+/CD8+ ratio, hold significant value for risk stratification and prognosis in DLBCL. We propose that integrating this ratio with the IPI could refine risk assessment, and its dynamic monitoring may guide personalized therapy. Prospective validation is warranted to confirm these findings and advance their clinical translation.

## Data Availability

The raw data supporting the conclusions of this article will be made available by the authors, without undue reservation.
